# Genome-wide screening of lectin putative genes from *Sorghum bicolor* L., distribution in QTLs and a probable implications of lectins in abiotic stress tolerance

**DOI:** 10.1186/s12870-022-03792-6

**Published:** 2022-08-13

**Authors:** Makarim El-fadil M. Osman, Amina Ibrahim Dirar, Emadeldin Hassan E. Konozy

**Affiliations:** 1 Department of Zoology, Faculty of Science, University of Khartoum, Khartoum, Sudan; 2 Medicinal, Aromatic Plants and Traditional Medicine Research Institute (MAPTRI), National Center for Research, Mek Nimr Street, Khartoum, Sudan; 3 Department of Biotechnology, Africa City of Technology, Khartoum, Sudan

**Keywords:** Sorghum, Lectins, QTLs, Domain architectures, Lectins expansion, Tolerance, Resistance

## Abstract

**Background:**

*Sorghum bicolor* is one of the most important crops worldwide with the potential to provide resilience when other economic staples might fail against the continuous environmental changes. Many physiological, developmental and tolerance traits in plants are either controlled or influenced by lectins; carbohydrate binding proteins. Hence, we aimed at providing a comprehensive in silico account on sorghum’s lectins and study their possible implication on various desired agronomical traits.

**Results:**

We have searched sorghum’s genome from grain and sweet types for lectins putative genes that encode proteins with domains capable of differentially binding carbohydrate moieties and trigger various physiological responses. Of the 12 known plant lectin families, 8 were identified regarding their domain architectures, evolutionary relationships, physiochemical characteristics, and gene expansion mechanisms, and they were thoroughly addressed. Variations between grain and sweet sorghum lectin homologs in term of the presence/absence of certain other joint domains like dirigent and nucleotide-binding adaptor shared by APAF-1, R-proteins, and CED-4 (NB-ARC) indicate a possible neofunctionalization. Lectin sequences were found to be preferentially overrepresented in certain quantitative trait loci (QTLs) related to various traits under several subcategories such as cold, drought, salinity, panicle/grain composition, and leaf morphology. The co-localization and distribution of lectins among multiple QTLs provide insights into the pleiotropic effects that could be played by one lectin gene in numerous traits.

**Conclusion:**

Our study offers a first-time inclusive details on sorghum lectins and their possible role in conferring tolerance against abiotic stresses and other economically important traits that can be informative for future functional analysis and breeding studies.

**Supplementary Information:**

The online version contains supplementary material available at 10.1186/s12870-022-03792-6.

## Introduction

Sorghum (*Sorghum bicolor* L. Moench; family: Poaceae) is the second key food staple crop in Africa after corn, as well as the fifth produced grain worldwide after maize, rice, wheat, and barley [1]. Sorghum gains its importance from its many agronomical features; such as drought tolerance, waterlog, and low demands for fertilizers [2, 3]. And the fact that it is a gluten-free cereal, which makes it a favorable source of nutrition in gluten-intolerant populations [4]. The plant originated in Northeast Africa, where Sudan and Ethiopia are recognized as the major center of its diversity [5]. Both the wild and cultivated species of *Sorghum* are genetically very diverse with 22 known species. There are five interfertile races (*bicolor, guinea, kafir, durra, and caudatum*), though *S. bicolor* L. is considered the major contributor to a wide range of cultivated sources. The plant grows in semi-arid tropics and is adapted to lower rainfall and high temperature (average between 24 – 27 °C after germination), which makes it a viable choice for a sustainable future, and production under climate change and global temperature increase. Nonetheless, the current global production is hampered by the low investments, climate constraints in regions with a wetter environment, and possible low yield [1, 6, 7].

Over the years, the integrated approach that combines agronomic research and plant breeding had successfully increased plant productivity and tolerance. One of the most effective tools used by plant breeders to study and incorporate a specific genetic trait is the quantitative trait loci (QTL) mapping, which is deployed for marker-assisted selection to identify genes responsible for certain traits [8]. Since 1995, around 150 QTL and genome-wide association studies (QWAS) were published related to sorghum, which is yet to be fully utilized in sorghum genetic research and enhancement programs. This is mainly due to the large heterogenicity in sorghum QTL’s data in terms of locations, maps used, reliability of many QTL experiments, and the variable sorghum breeds used across studies. These obstacles were overcome by the availability of sorghum whole genome sequence, genetic linkage consensus maps, and the use and availability of genetic markers coordination information. These data are now integrated at the Sorghum QTL Atlas (https://aussorgm.org.au/), which provided an accessible source of information for plant breeding programs. The atlas incorporates over 6000 QTL and GWAS linked to 223 unique traits that are classified under 7 major trait categories (leaf, maturity, panicle, resistance abiotic, resistance biotic, stem composition, and stem morphology) [9, 10].

Identifying and linking genes to certain QTL can be sometimes a laborious process. However, there are several ways to achieve the desired outcome. For instance; using the fine-mapping approach that narrows the QTL to a specific genomic region and then to a specific gene or several genes. For instance, this approach successfully correlated the *ZmCCoAOMT2* gene that encodes caffeoyl-CoA *O*-methyltransferase to the qMdr_9.02_ QTL which attributes to the resistance of grey leaf spot and southern leaf blight in maize [11]. The QTL mapping – expression eQTL analysis is also a method directed towards identifying candidate genes underlying or associated with multiple QTL, for example; the A10 QTL from *Brassica napus* contains the *BnaA10g22080D* gene that modulates the regulation of flowering time [12]. The availability of full genome sequences and assemblies made it possible for the use of bioinformatics tools to correlate QTLs with the occurrence of specific genes. it was used to study the distribution of the Receptor-like cytoplasmic kinase gene family (*OsRLCKs*) within the abiotic stress QTL in rice, and examine the distribution and overrepresentation of lectin families in morphological traits and stress responses [13, 14]. Many studies highlighted the association between lectins and QTL, for example, the MLOC_72613 gene encodes the L-type lectin domain-containing receptor kinase III which regulates water status under drought stress through the regulation of the brassinosteroid mediated pathway in barely [15]. In sorghum grown under different nitrogen levels, two lectin genes are upregulated (Sb01g033090, Mannose-binding lectin superfamily, and Sb01g045620, lectin protein kinase family protein). The mannose-binding lectin is associated with the QTLs related to the stover moisture content (qMC2-1a), the head moisture contents (qMC2-1), and the total biomass yield (qBY-1), whereas lectin protein kinase is correlated to the grain yield QTL (qGY-1b) [16].

Lectins are a heterogeneous group of proteins capable of binding reversibly to carbohydrate moieties of simple or complex glycans, with different affinity and avidity, and without altering their chemical structures [17]. Initially, lectins were believed to be localized in extracellular compartments and only expressed in response to a foreign organism as defense molecules. However, evidence showed that they can interact with endogenous glycan-receptors found in the nuclear/cytoplasmic cellular compartments. Hence, playing a huge number of physiological functions [18]. Due to restricted mobility, plants are unable to circumvent harsh environmental and unfavorable conditions. Consequently, they have to acclimatise to such situations by changing many of their physiological and molecular responses as well as regulating their metabolic pathways [19, 20]. Besides being involved in many plant physiological significances such as defense against predators [21], storage protein [22], in-cell sugar transport [23], etc., the role of plant lectin in different cellular locations as a stress adaptor has also been explored in different crops [24, 25]. For example, oil seed flax lectin was interestingly shown to possess different sugar specificities under different stress conditions, in addition to this, the lectin coefficient activity i.e., (1/lectin activity) was also noticed to vary considerably based on the type of stress and lectin organ of localization, authors have attributed these fluctuations in sugar specificity and lectin activity to stress modulating role of lectin [26]. At the transcriptomics level, rice (*Oryza sativa*) expresses 5 lectin genes (OsEULS2, OsEULS3, OsEUL1a, OsEUL1b, and OsEUL2D). Treatment of the plant with different stress types (biotic or abiotic) results in the detection of different EULs transcripts with variable levels of expression. Most of which were detected in the vascular tissues of roots and shoots, as well as the root tips and seeds [27]. The transgenic *Arabidopsis thaliana* plant harboring Nictaba-Like Lectin genes from *Glycine max* exhibits enhanced tolerability against bacterial infection compared to the wild-type plant. These outcomes indicated the involvement of *G. max* lectin in the biotic stress tolerance [25].

Numerous classification systems have been adopted for lectins, which included grouping them based on their sugar specificity, the structure of the mature protein, the primary sequence similarities, and their domain architectures. Genomic-wide screening and domains studies of lectin homologs showed that the majority of lectin putative genes are chimeric lectins, constructed by fusing a single or multiple lectins domains to another protein domain. This allows lectin homologs that belong to the same family to function in diverse physiological pathways [28–30]. Despite the wealth of information about plant lectins in terms of physicochemical, structural, expressional, and genomic studies, as well as their possible application [31–34], there had been no attempt to isolate and study sorghum lectins. To the best of our knowledge, only a single study was published in 1982; where seed lectins from 5 sorghum species were isolated and marginally characterized with respect to blood and sugar specificity only [35].

In this study, we investigated the presence, distribution, and evolution of lectin homologs predicted from the *S. bicolor* genomes (grain and sweet). Moreover, their occurrence in sorghum QTLs in relation to several traits such as biotic and abiotic stress and tolerance, morphological, composition, and maturity traits were also investigated. This work can provide valuable insights on sorghum’s QTL regions, and lectins’ physiological roles related to plant defense/tolerance, growth, and development.

## Results

### Identification and distribution of lectin homologs from grain and sweet sorghum

**Fig. 1:**
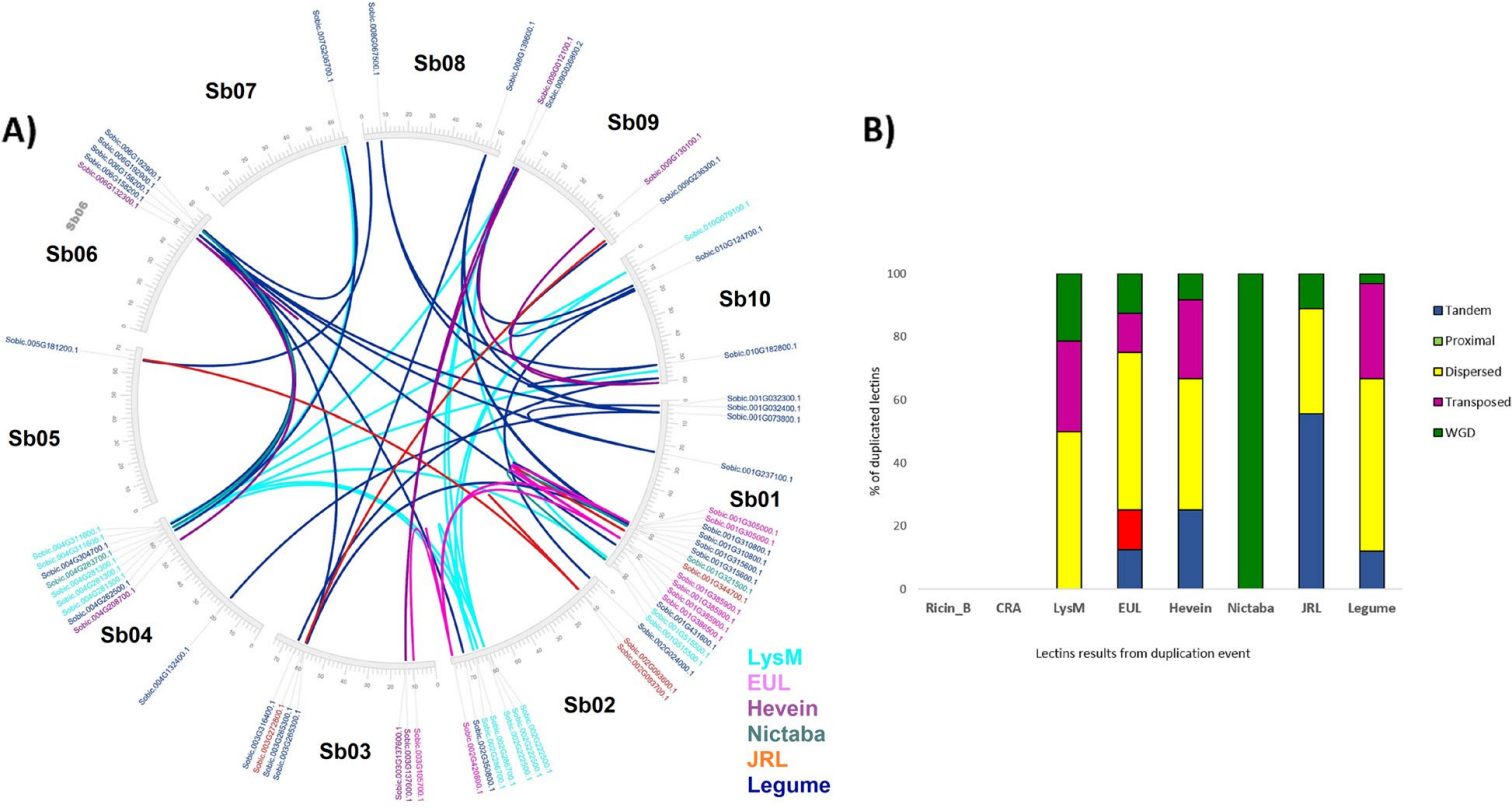
Putative lectin gene homologs duplication from grain sorghum V3.1. **A** Segmental duplication map, **B** the evolutionary events responsible for each lectin family expansion

Two genetically variable types of *Sorghum bicolor* genome assemblies, i.e., grain (BTx623) V3.1 (732.2 MB), and sweet (Rio) V2.1 (729.3 MB), deposited in the Phytozome V13 were screened for lectin gene homologs [36, 37]. A total of 119 and 113 genes, respectively, were extracted. Of the 12 known plant lectin families, 8 families were identified upon searching both sorghum genomes. They are Ricin-B, CRA, LysM, EUL, Hevein, Nictaba, JRL, and leg-B. Around 45% of the lectin homologs are from the legume family followed by the jacalin-related lectins (15%) and the Nictaba homologs (14%) (Table 1). The total number of chimeric putative lectins in grain sorghum is up to 83 genes (70%), and 81 genes (72%) in sweet sorghum. *Sorghum bicolor* has a haploid chromosomal number of 10 chromosomes. The distribution and abundance of each lectin family and its putative genes are heterogeneous with very minor variations between the two races. In general, lectin genes are clustered in both chromosomes 1 and 2 (36% of the total genes), while chromosomes 7 and 8 had the least number of genes, all of which are related to legume and LysM families (Additional file 1: Figure S1). 44% of the total lectin-related genes were subjected to different expansion events, dispersion and transposition events are the main cause of the genes duplication and contribute to up to 70% of the total lectin homologs’ expansion. However, 56% of the expanded Jacalin-related lectins were tandemly duplicated, while the only 2 Nictaba-related putative genes subjected to duplication were retained after a wide genome duplication event (WGD) (Fig. 1).

**Table 1:** Predicted lectin homologs from the grain and sweet Sorghum bicolor genome assemblies

Race	Lectin Domains	Predicted Genes		Chromosomes Localization	Predicted Signal Classes ^a^
		**No**	**%**		
*S. bicolor* V3.1 (BTx623)	Ricin-B	2	1.7	2, 5	UPS (1), SP (1)
	CRA	2	1.7	5, 6	SP (2)
	EUL	8	6.7	1, 2, 3, 5	USP (4), IC (3), SP (1)
	Hevein	11	9.2	3, 4, 6, 9, 10	SP (11)
	Nictaba	17	14.3	1, 2, 3, 4, 5, 6	USP (6), IC (10), TM (1)
	JRL	18	15.1	1, 2, 3, 5, 9	USP (8), IC (8), TM (2)
	Legume	53	44.5	1, 2, 3, 4, 5, 6, 7, 8, 9, 10	USP (1), IC (2), TM (5), SP (45)
	LysM	8	6.7	1, 2, 3, 4, 9, 10	TM (1), SP (7)
*S. bicolor* V2.1 (Rio)	Ricin-B	2	1.8	2, 5	USP (1), SP (1)
	CRA	2	1.8	5, 6	SP (2)
	EUL	9	7.9	1, 2, 3, 5, 9	USP (5), IC (2), SP (2)
	Hevein	10	8.8	3, 4, 6, 9,10	SP (10)
	Nictaba	17	15	1, 2, 3, 4, 5, 6	USP (7), IC (9), SP (1)
	JRL	18	15.9	1, 2, 3, 5, 9	USP (7), IC (10), TM (1)
	Legume	53	46.9	1, 2, 3, 4, 5, 6, 7, 8, 9, 10	USP (1), IC (4), TM (5), SP (43)
	LysM	2	1.8	2, 7	SP (2)

^a^ *TM* Transmembrane, *SP* Signal peptide, *IC* Intercellular and *UPS* unconventional protein secretions (UPS) from intracellular proteins, which do not possess either a signal peptide or transmembrane domain

### Domain architecture and characterization of lectin homologs

**Fig. 2:**
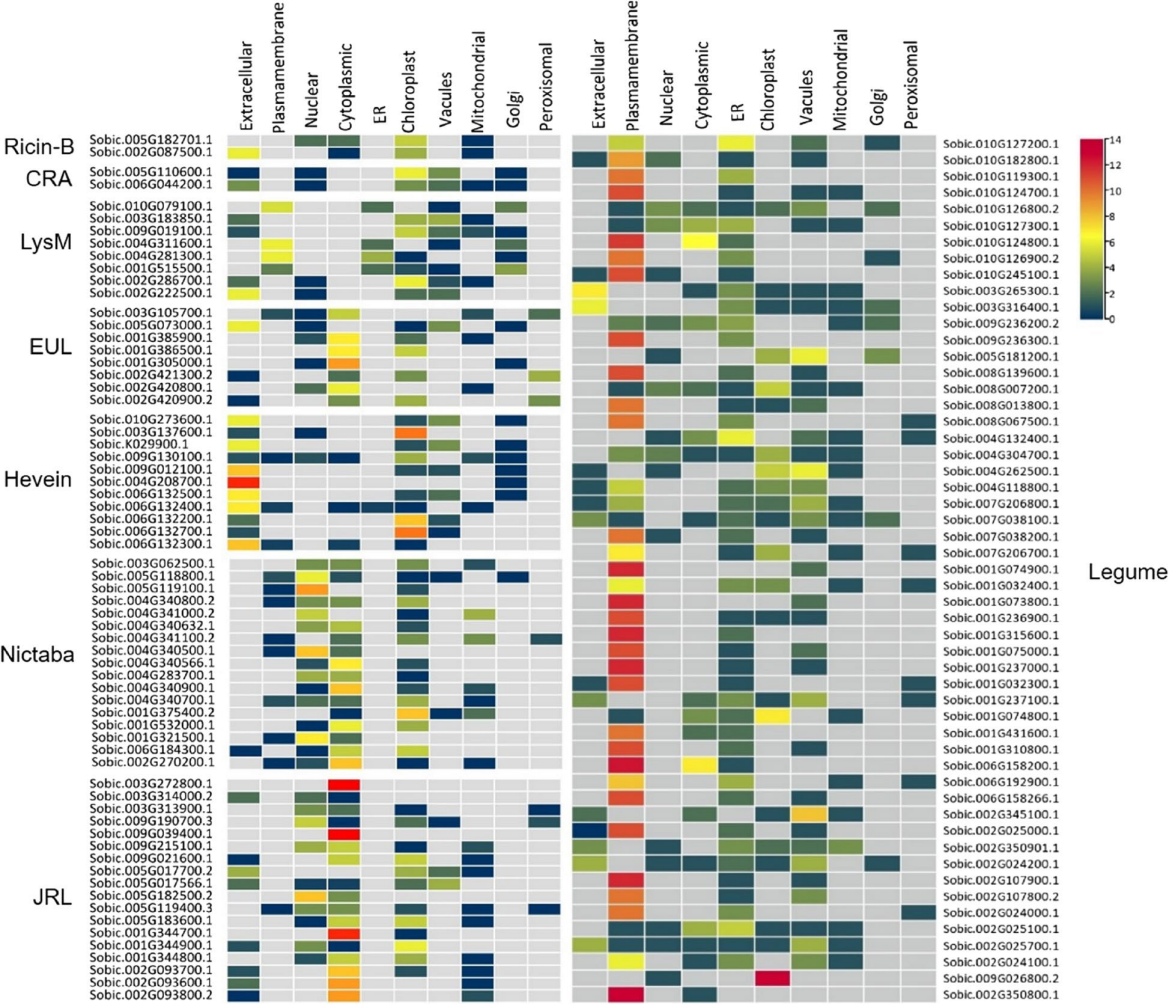
Prediction of grain sorghum’s putative lectin genes subcellular localization

**Fig. 3:**
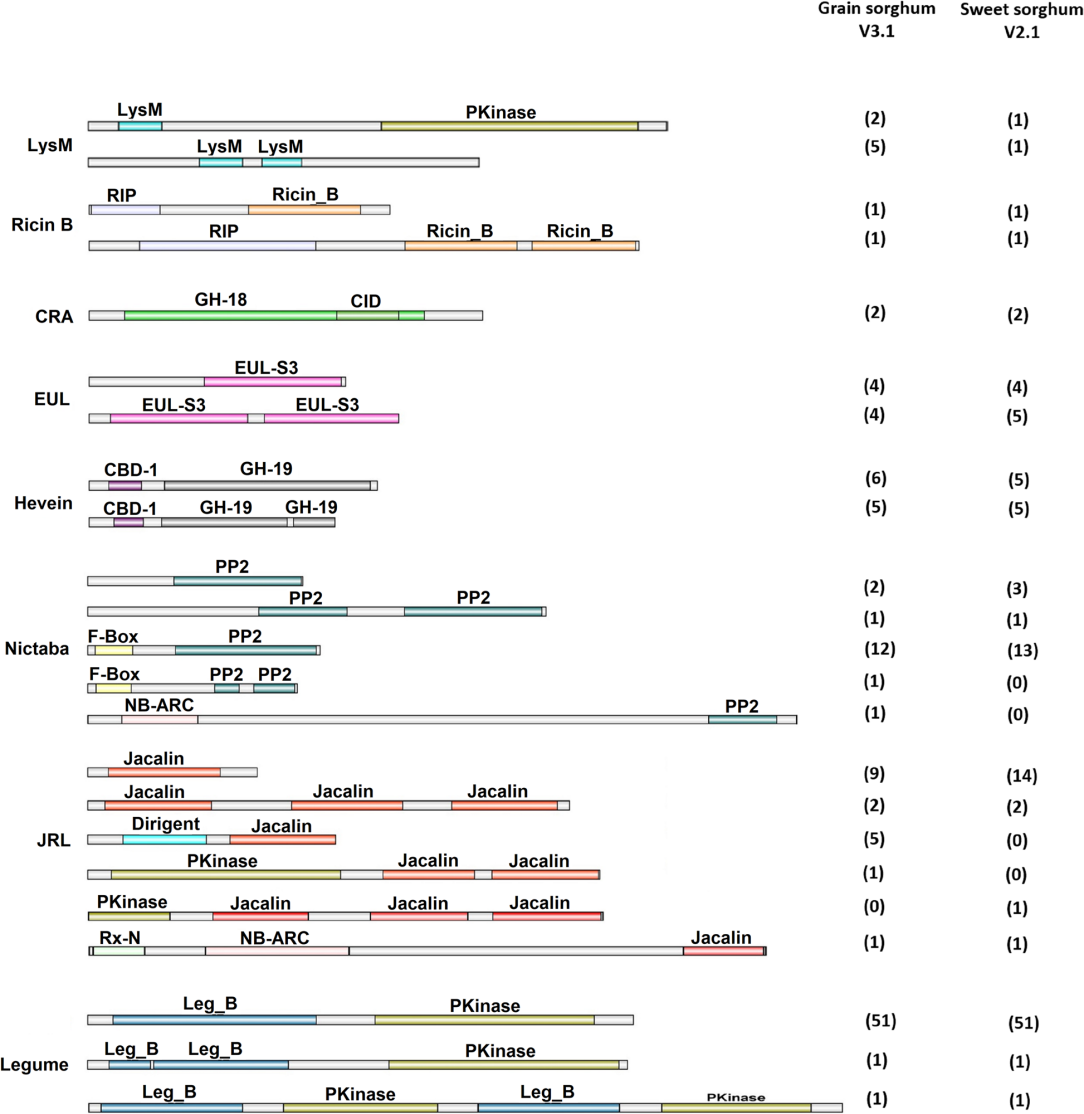
Representation of domain architecture and abundance in grain and sweet sorghum. LysM: Lysin motif, GH-19: glucosyl hydrolase-19 (PF00182), CBD-1: carbohydrate binding domain-1, PP2: Phloem protein 2, F-box (PF00646), NB-ARC (PF00954), Dirigent (PF03018), Rx_N (PF18052), PKinase: Protein kinase (PF00069, PF07714)

Sorghum putative lectin homologs are structurally organized into three groups; merolectins of a protein with a single lectin domain, hololectins where two or more lectin domains are linked, and chimerolectins that are constructed from a single or multiple lectin-domains fused to a single or multiple other protein domains [38] (Fig. 2, Fig. 3, and Additional files 2: Table S1, and Table S2). Ricin-B lectin homologs are glycosylated chimeric-proteins (~ 35 – 65 kDa) that belong to the type-II ribosomal inactivating proteins. The putative gene is built from a single ribosomal inactivating domain (RIP) that has RNA *N*-glycosidase activity (Pfam: PF00161), and forms the A-chain of the protein; fused by the C-terminal to either a single or two tandemly arrayed carbohydrate-binding domain Ricin-B that forms the B-chain. Only the double Ricin-B domains protein, which is constructed from a single exon, is predicted to be a secreted lectin with a signal peptide sequence attached N-terminally to the RIP domain. Sorghum’s class-V chitinase-related agglutinin genes (CRA) are exclusively merolectins that contain a short sequence of chitinase insertion domain (CID) of approximately 70 amino acids. All genes that are structured from a double exon and a single intron, have signal peptide sequences, devoid of the transmembrane domain, and are most likely to be targeted to the chloroplast, cytoplasm, or extracellularly. Like all monocots, the EUL homologs are either raised from a single or double lectin domain that belongs to type S3, which is proceeded by a long unrelated N-terminal sequence (~ 17 – 48 kDa). Most EUL lectins are synthesized without a signal peptide, hence they are considered nucleoplasmic proteins. However, one sequence from grain sorghum and two sequences from sweet sorghum have a signal peptide and are predicted to be targeted to the vacuoles and/or the chloroplast. Sorghum legume and Hevein families are both strictly chimeric lectins. Hevein is a monomer protein (~ 28 – 38 kDa) that binds a homopolymer of *N*-acetyl-D-glucosamine. A sole lectin domain is linked through the C-terminal to one or two domains of glycosyl hydrolase-19 (GH-19) that belong to the class-I chitinase group. 7 of the Hevein-like genes located at both chromosomes 6 and 9 contain a short intron sequence of approximately 88 bp. They are secreted proteins targeted either extracellularly or to the chloroplast [38]. Lectin-receptor kinases (Lec-RKs) are the most abundant type of lectins, which in sorghum encompass all putative genes from the legume lectin family, members of the LysM, and Jacalin-related families. Compared to legume homologs, LysM and Jacalin-RKs genes are structurally heterogenic in terms of their exon/intron length and number. In legumes and LysM Lec-RKs putative homologs, the protein kinase domain is proceeded either by the leg-B domain or the lysin motif, respectively. This is reverse to the architecture of the Jacalin-PKinase gene, where the tandemly arrayed Jacalin domains are proceeded by the Kinase domain. Unlike the LysM and Legume Lec-RKs homologs, the jacalin-PKinase is devoid of the signal peptide and the transmembrane domain, hence considered a nucleoplasmic protein. Other protein domains were reported to be linked to the JRL domains, such as the dirigent, NB-ARC, and Rx_N motifs. They are mainly found in proteins related to disease response in plants [39, 40]. The NB-ARC domain is also found fused to a member of the Nictaba family from only the grain sorghum race (179 kDa). Moreover, the sweet sorghum genome also lacks the jacalin-related lectins that contain the dirigent domain (Additional file 1: Figure S2).

### Distribution of lectin sequences within QTLs from grain sorghum

**Fig. 4:**
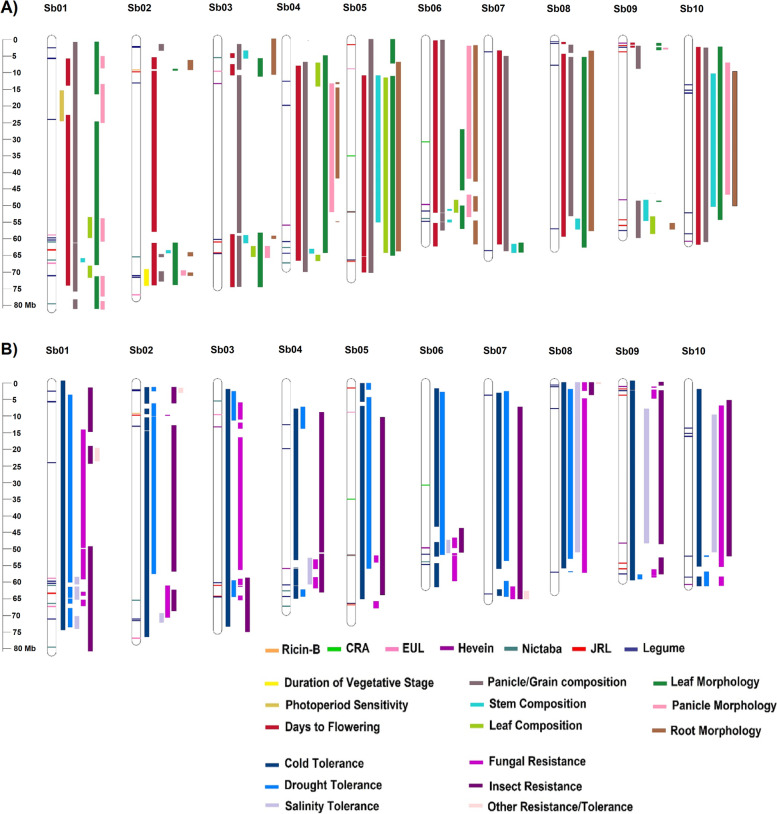
Grain sorghum chromosomal physical map with lectin distribution and QTL regions. **A** Mapping QTLs for sub-categories related to maturity, composition, and morphology. **B** Mapping QTLs related to abiotic and biotic tolerance/resistance

**Fig. 5:**
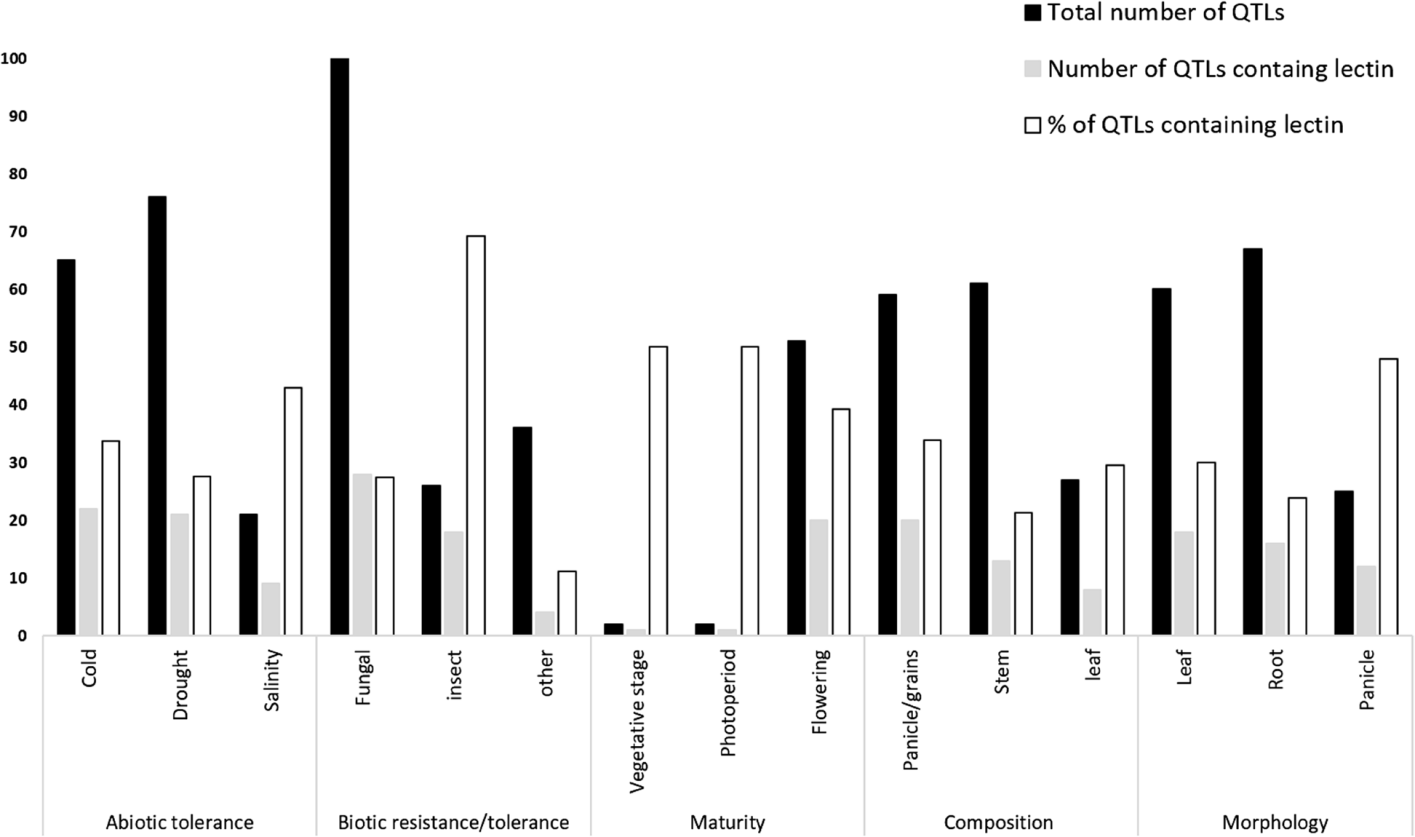
Total number of QTLS for sub-categories and the number and percentage of QTLs with lectin genes

We screened the grain sorghum genome for lectins' wide distribution and association with QTL regions. Physical mapping of QTLs to sorghum genome’s (V3) 10-chromosomes and their subsequent intersections with lectins revealed that 96.6% of the total number of lectin sequences are located within one or more QTL regions of all major categories. Merging the QTLs of 223 unique traits into non-redundant unique QTLs under 9 sub-categories of 5 major categories (abiotic, biotic, maturity, composition, and morphology) resulted in stretching the QTL regions of several sub-categories all over certain chromosomes like 5, 8, and 10 (Fig. 4). Mainly, due to either the higher number of QTL in traits under question or the large size of these traits’ QTL. Only 34.3% of these QTLs contain lectin genes (Fig. 5). And unlike members of lectin families (Ricin-B, CRA, EUL, Hevein, Nictaba, and JRL) which are distributed in 6 out of 9 sub-categories, members of LysM and legume families are present in all of them (Additional files 3, and 4). Statistical analysis indicated that lectin sequences are only preferentially distributed in QTL regions related to root morphology under the major category morphology (*z* score = -1.8905, *P*_*0.05*_ = 0.029345). However, the analysis of all unique traits with QTL regions containing lectin genes (123 out of 223 traits), showed that several traits related to abiotic stress, panicle/grain composition, and morphology of the leaf contain significant lectin putative genes overrepresentation (Additional file 5).

#### Lectin distribution in QTLs related to maturity, composition, and morphological traits

Lectin sequences are distributed in maturity quantitative trait loci with a percentage between 50 and 100%. However, they are primarily clustered in QTL regions associated with the days to a flowering trait with a total of 84 genes from all reported lectin families. A dirigent-JRL *VER2* gene from *Triticum aestivum* which is homologous to Sobic.009G021600.1 and Sobic.005G183600.1 genes (sharing the identity of 40.66 and 39.19%, with both genes, respectively) located in the QTL-days to flowering was reported to mediate flowering upon inducing vernalization of seeds during germination. Knock out of the *VER2* gene resulted in the delay of flowering for up to 6 weeks [41]. 83% of the total lectins are located in traits related to panicle/grain composition, with about 47 Lec-RK genes from the legume family. The significant overrepresentation of lectins was reported in composition category under QTLs related to grain element concentration and embryo size (z score = -2.064867, P0.025 = 0.019468, z score = -2.21837, P0.025 = 0.013252, respectively). Although there is no significant preferential representation in traits under the stem and leaf composition subcategories, lectin putative genes are concentrated in QTLs associated with stem cellulose and hemicellulose content, as well as leaf chlorophyll content (20, 9, and 12%, respectively). GRMZM2G402417 dirigent-JRL lectin identified in *Zeya maize* is closely related to other members of grass plants including sorghum, is upregulated and expressed up to 105-fold in grass-related cell-wall processes [42], its counter gene homolog in sorghum Sobic.009G021600.1 is located in a QTL related to cellulose content (stem composition). In morphological traits, 65 genes (58.6%) were found in leaf morphology QTL, whereas 30.6% and 32.4% were reported for panicle/grain and root morphology characteristics. Though, Tsaneva and colleagues reported that the QTLs for all morphological traits in *Oryza sativa* did not contain any lectin sequence from the families CRA and EUL [14, 43], in grain sorghum members of the EUL and CRA-related lectins can be found in QTLs of leaf and panicle morphological traits. The CRA gene Sobic.006G044200.1 located in QTLs associated with leaf width and angel, panicle length, and root brace was reported as one of the yieldin gene group responsible for the cell wall loosening associated with assembly and degradation [44]. (Additional files 3, 4, and 5).

#### Lectin distribution in QTLs related to abiotic and biotic tolerance/resistance traits

The biotic and abiotic QTLs which are associated with conferring resistance or tolerance during stress conditions comprise about 47.9% of the total QTLs found in the sorghum QTL atlas. All 36 traits under the cold and drought sub-categories contain at least one lectin gene from all families with significant overrepresentation in leaf growth rate (*z* score = -2.5704998, *P*_*0.025*_ = 0.005078) and germination index (*z* score = -1.7593058, *P*_*0.05*_ = 0.039263). Up to 48 lectin sequences are found in QTLs related to dry matter growth (cold tolerance sub-category) with 61% of the JRL members and 32% of Legume-like genes. Sobic.003G105700.1 gene is a member of the EUL family located at chromosome 3. Functional enrichment analysis using STRING predicted that its interacting partner is the embryo-specific protein (ATS3A). This lectin is a homolog to ArathEULS3 (At2g39050) from *A. thaliana* with a shared identity of 68%. ArathEULS3 has been linked to drought stress response and is believed to play a role with its partners ATS3A and ATS3B in ABA-induced stomatal closure [45]. Sobic.003G105700.1 is located in QTLs related to stay-green, and chlorophyll content traits, which are considered very important QTLs linked to drought and cold stresses [46]. Moreover, 39% and 47% of the total putative lectin genes contain a varying number of cis-acting elements that modulate gene expression during low temperature (LTR element) and drought (MBS element), respectively. Abscisic acid (ABA) is a plant hormone that coordinates different stress signals. Under low temperature and dehydration, the plant triggers ABA secretion, which results in the release of many transcriptional factors [47]. About 89% of sorghum lectin putative genes are either up- or down-regulated by ABA through the ABRE-cis-acting element (Additional file 6).

In biotic resistance traits, the highest number of lectin homologs were found in QTLs that connected to insect resistance (70 lectins putative genes), followed by fungal and parasitic resistance traits (64, 14 lectins, respectively). In fungal-related QTLs, lectins are concentrated in QTLs associated with rust resistance, and ergot resistance (% of infection and pollen quantity). While in insect-related QTLs, lectins are mainly located in green bug resistance, shot fly resistance, and head bug resistance-related loci. Although there was no significant overrepresentation of lectins in QTLs related to biotic resistance, many lectins’ promoters were reported to have cis-acting regulatory elements related to elicitation, wounding and pathogen responsiveness (20% box S and WUN-box, and 49% W-box) (Additional file 6). Furthermore, several protein domains are reported to be involved in plant immunity and defense against biological predators and parasites. For instance, NB-ARC [40], dirigent [48], and protein kinases [49] are domains that can be found fused to one or more lectin domains such as jacalin, Nictaba, LysM, and legume domains [21]. Interestingly, unlike the grain sorghum, the genome of sweet sorghum is devoid of the dirigent-JRL sequences compared to the 5 genes that are found in grain sorghum. However, these dirigent-JRLs have an evolutionary relationship with three single domain JRL putative genes from sweet sorghum (SbRio.02G096700.1, SbRio.02G096800.1, and SbRio.02G096600.1) (Fig. 3, Additional file 1: Figure S2). A dirigent-jacalin sorghum lectin (SL), which is a homolog to the β-glucosidase aggregating factor (BGAF) from maize, is suggested to mediate insects and pathogens interactions by lectin activity rather than modulating β-glucosidase [50] (Additional files 3, 4 and 5).

## Discussion

Sorghum is an important economic C_4_ crop with a simple genome, diverse phenotypes (i.e., grain, sweet, forage, and cellulosic sorghums), and water-use efficiency that allows it to produce under adverse conditions and low levels of required inputs [51]. Studies demonstrated that the phenotypical variation between grain and sweet sorghum is a reflection of the genetic differences which occur due to gene variation (~ 1,500 genes), SNPs, insertion/deletion (indels) segments, and the presence/absence variations (PAVs) in genetic regions. The majority of these genetic variations reside in genes with adverse biological functions and stress responses [52–54]. This can be noted in the variation observed in the simple sequence repeats (SSR) found in some of the lectins genomic sequences interms of numbers, length and sequence composition, and can be used for plant selection and identification of domesticated cultivors [55]. Moreover, there is a variation in lectin gene numbers observed for each family, especially the LysM family. Plant LysM-like lectins are chitin-elicitor proteins, which are involved in cell surface pattern recognition receptor signaling pathways leading to innate immunity against biotic (bacterial and fungal pathogens) [56], and abiotic stresses (tolerance to salinity, and heavy metal stresses) [57–59].

On the other hand, genetic duplication in sorghum is relatively low compared to other grass-member crops like maize and rice. *Oryza sativa* genome is bigger than sorghum’s genome and went through more gene expansion events than sorghum [60]. Although the number of lectin genes homologs in rice are triple the gene size of sorghum, the majority of rice lectin genes located in nonsegmental regions were expanded through tandem duplication events [14], whereas around 85.8% of sorghum’s lectin genes were expanded by various segmental events compared to the 16.6% tandemly duplicated lectin genes, mainly by dispersion or retrogene copies created by transposition event. The variation in genes number in each lectin family is also an indication that each family has evolved differently. Members of CRA and Ricin-B families had been retained in the sorghum genome without any duplication event, meanwhile, the rest of the lectin families evolved through various duplication mechanisms (either tandem or segmental or both) and were preserved through neofunctionalization or sub-functionalization events that offered various biological and physiological needs or adaptive benefits against environmental stresses. While there is conservation in the exon/intron organization between lectin gene homologs from the same family in general, many duplicates diverge from the common structure either by insertion (e.g. Sobic.001G386500.1-EUL and Sobic.002G420800.1-EUL duplicated by dispersion), or deletion (e.g. Sobic.003G313900.1-JRL and Sobic.003G314000.2-JRL tandemly duplicated) or both. The variation observed in intron number and length between gene members of the same family and between the different lectin families suggests functional roles related to splicing, enhancement of gene expression, controlling mRNA transport or chromatin assembly, and providing a source for new genes [61, 62]. On the other hand, the diversity observed in lectin putative genes architectures and rearrangement in terms of lectin domain number and/or fusion with other protein domains seen in lectin families and between members of the same family from sweet and grain sorghum, especially JRL, Nictaba, and EUL can be explained by key mechanisms related to a single-step terminal loss, fusion or fission [63].

Understanding the endogenous physiological role of plant lectins depends on several elements including domain arrangement, overall 3D structures, carbohydrate specificity, ultrastructural cellular location, interacting partners within pathways, and their differential expressional pattern during the life cycle of the plant. The presence and absence of signal peptide and/or transmembrane domains within members of sorghums’ same lectin family or between the different families indicate that putative lectins will end up in different cellular compartments, and eventually enters different pathways. Studies showed that plant lectins can physiologically function as plant innate immunity defense molecules, symbiotic receptors for microorganism attachment, pollen recognition, storage protein, and in various signal transduction pathways related to cellular organization, cell-wall elongation, embryogenesis, sugar transportation, wound healing, etc. [18, 21, 64, 65]. Although we’ve identified 119 and 113 lectin putative genes in grain and sweet sorghum respectively, there is no account for the isolation and extensive characterization of lectins from sorghum. The only paper published attempted to test the agglutination activity and blood specificity of sorghum whole grain crude extract [35]. However, several transcriptomic and expression analysis data that mentioned several sorghum lectins, as well as identified many lectin genes in QTLs of many traits are available [14, 16, 66, 67]. The statistically significant overrepresentation and clustering of sorghum’s lectin putative genes in QTLs related to many traits associated with drought, cold, composition, and morphology highlights the importance of such proteins, and predominantly associates them with plant developmental and growth aspects as well environmental adaptation.

Several lectins from grass members were functionally characterized in association with many traits. The endophytic colonization of *Herbaspirillum seropedicae* (a plant growth-promoting bacteria associated with crops like maize, sorghum, rice, and wheat [68]) to maize roots is mediated by jacalin-related lectins (MRL-1 and MRL-2) interaction with the diazotrophic betaproteobacterium lipopolysaccharides’ *N*-acetyl glucosamine residues [69]. A Ricin-B-like lectin *TaRBL* from wheat is conferring resistance to Fusarium head blight (FHB) fungal pathogen *Fusarium graminearum* by physically interacting with the pore-forming toxin-like protein *TaPFT* (its gene located in the quantitative trait locus Fhb1 associated with the Fusarium resistance). During the infection, the *TaRBL* expression is upregulated in resistant cultivars and downregulated in the susceptible ones. Furthermore, inducing *TaRBL* gene silencing in resistant cultivars using stripe mosaic virus resulted in an apparent reduction of FHB resistance [70].

Transcriptomic analysis of sweet sorghum stem expressed genes indicated that lectins genes are involved in the carbohydrate metabolism of stem sugars, where a mannose-binding lectin Sb10g022730.1 and LysM lectin Sb01g049890.1 with possible involvement in cell-wall degradation were twofold upregulated [71]. As stated in our results, the Sobic.006G044200.1 CRA putative gene located in major traits subcategories related to the morphology of leaf, panicle, and roots (leaf width and angle, panicle length, and brace roots) was reported to function in cell wall loosening [44].

Co-localization of QTLs between traits is often associated with differential gene expression. Several lectin genes are expressed under various nitrogen level stresses. Sb01g033090 sorghum gene belongs to the mannose-binding lectin superfamily is expressed with a fourfold increase in normal nitrogen levels under QTLs associated with the % stover moisture content (qMC2-1) and % head moisture content (qBY-1), whereas the expressional levels of a lectin with LysM domain (Sb01g048100) decreased up to 4.1-fold in the biomass yield QTL (qGY-1b) that partially overlaps with qMC2 and qBY-1 loci [16]. Since adaptation to environmental and climatic changes is often associated with physiological and morphological changes it was only natural to find the majority of the sorghum lectins located in QTLs of such traits to overlap with other QTLs related to biotic and abiotic stresses’ tolerance/resistance. In rice, OslecRK a receptor-kinase lectin provides the genetic evidence that links seed germination and plants innate immunity. Knocking out the OslecRK led to the down-expression of α-amylase genes which resulted in plummeting seed viability and subsequently decreasing the rate of germination, as well as reducing plant resistance to bacterial, fungal, and insect pathogens [72].

## Conclusion

In conclusion, while there is a scarcity of knowledge on sorghum lectin studies in terms of biochemical and functional analysis compared to other grass members especially rice and maize, a glimpse of possible physiological roles was highlighted in other expressional studies among other genes. Lectins are at the center of plant developmental and adaptational events. Understanding their particular functions as well as possible pleiotropic effects within certain pathways, and how they contribute to various traits can offer tangible insights that help to produce more resilient cultivars with desired phenotypical traits. Our results provide a collective overview of sorghum lectins from grain and sweet types and the microvariations between members of lectin families from both types. The distribution of grain lectin putative genes homologs in various QTLs and their significant overrepresentation in certain traits related to cold and drought tolerances can provide valuable information that may contribute to the production of abiotic resilient plants in times that other crops like rice are extremely challenged with the global temperature increase.

## Methodology

### *Sorghum bicolor* genome screening for putative lectin genes

The genomic assemblies of *Sorghum bicolor* (BTx623 V3.1.1; ID:454, and Rio V2.1; ID:468) [36, 37] found in the public plant genomic resources repository Phytozome V13 (https://phytozome-next.jgi.doe.gov/) [73] were screened against the 12 known plant lectin families identified from *Agaricus bisporus* agglutinin (ABA – UniProtKB/SwissProt: Q00022.3, Pfam: PF07367), *Amaranthus caudatus* agglutinin (amaranthin – GenBank:AAL05954.1, Pfam: PF07468), *Nostoc ellipsosporum* agglutinin (cyanovirin—UniProtKB/SwissProt: P81180.2, Pfam: PF08881), *Euonymus europaeus* agglutinin (EUL – GenBank: ABW73993.1 Pfam: PF14200), *Galanthus nivalis* agglutinin (GNA – UniProt/SwissPRot: P30617.1, Pfam: PF01453), *Artocarpus integer* agglutinin (JRL – GenBank: AAA32680.1, Pfam: PF01419), *Glycine max* agglutinin (Legume – UniProt/SwissProt: P05046.1, Pfam: PF00139), *Brassica juncea* agglutinin (LysM – GenBank: BAN83772.1, Pfam: PF01476), *Hevea brasiliensis* agglutinin (Hevein – GenBank: ABW34946.1, Pfam: PF00187), *Robinia pseudoacacia* agglutinin (CRA – GenBank: ABL98074.1, Pfam: PF00704), *Nicotiana tabacum* agglutinin (Nictaba – GenBank: AAK84134.1, Pfam: PF14299), and *Ricinus communis* agglutinin (Ricin-B – GenBank: PDB2AAI_B, Pfam: PF00652). Each agglutinin sequence was aligned against *S. bicolor* genome using the Phytozome-BLAST tool (E value < 0.0001). Sequences with the highest identity match were used to perform a second BLAST search, then the generated candidate lectins for each family were retrieved using the BioMart tool and each protein sequence was individually checked for the presence of at least one lectin domain using the InterProScan 5 (http://www.ebi.ac.uk/interpro/) [74].

### Putative gene structure and cis-acting elements analysis

The diversity and structure of lectin putative genes from each family (grain sorghum genome V3) were investigated by comparing the exon/intron organization of each gene coding sequence (CDS) and its corresponding genomic DNA sequence using Gene Structure Display Server GSDS (http://gsds.cbi.pku.edu.cn/) [75]. Furthermore, the promoter regions (1500 bp upstream of each genomic sequence of each gene) were searched for the presence of cis-acting elements using the webtool PlantCARE [76] (https://bioinformatics.psb.ugent.be/webtools/plantcare/html/). Seven categories were used to cluster the elements. Simple sequence repeats (SSR) markers found in putative lectins genomic sequences were also identified using Simple Sequence Repeat Identification Tool (SSIT) found in GRAMEME (Srrtool) [77] (https://archive.gramene.org/db/markers/ssrtool) see (Additional file 7).

### Putative lectin gene characterization

Each putative lectin homolog was analyzed for the presence of signal peptides and transmembrane domain using SignalP 5.0 server (https://services.healthtech.dtu.dk/service.php?SignalP-5.0) [78], and (https://services.healthtech.dtu.dk/service.php?TMHMM-2.0) webtool transmembrane domains (TMHMM 2.0), respectively [79]. The subcellular localization for lectin homologs was predicted using WoLF PSORT (https://wolfpsort.hgc.jp/) [80]. Furthermore, the calculation of the molecular weight and isoelectric points (*pI*) for each sequence was done using Expasy: Compute pI/Mw (https://web.expasy.org/compute_pi/) [81], and the prediction of the *N*- and *O*-glycosylation were also performed using the following servers NetNGlyc 1.0 [82] (https://services.healthtech.dtu.dk/service.php?NetNGlyc-1.0) and (https://services.healthtech.dtu.dk/service.php?NetOGlyc-4.0) NetOGlyc 4.0 [83], respectively.

### Gene duplication analysis

The Plant Duplicate Gene Database PlantDGD (http://pdgd.njau.edu.cn:8080/) [84] was used to identify gene duplicates for putative lectin genes and determine the type of duplication (i.e.; tandem, proximal, dispersed, transposed, and wide genome duplication). This was followed by using the TBtools V1.0986853 Ka/Ks calculator to calculate the synonymous substitution (Ks) for lectin genes and their duplicates, and values higher than 1 were excluded [85].

### Phylogenetic analysis of lectin homologs

Phylogenetic trees were constructed using only lectin domain sequences from each family. Sequences that contain more than one lectin domain were trimmed and each domain was used as a separate entry. Multiple sequence alignment Clustal W tool built on MEGA X software was used. The obtained files were used for the evolutionary analysis by the maximum likelihood method and the JTT matrix-based model of substitution. The final bootstrap consensus trees were inferred from 1000 replicates [86]. Genes related to each family from both *Arabidopsis thaliana* and *Orzya sativa* were used as orthologous groups for comparison.

### Functional enrichment analysis

Putative lectin genes were analyzed to identify the interacting partners of each gene using the protein–protein interaction network STRING 11.5 server (https://string-db.org/) [87].

### Mapping of lectin genes on chromosomes and QTL regions

QTL and their physical positions, linked groups, and description were retrieved from the OZ sorghum QTL Atlas database (https://aussorgm.org.au/) [10], and divided into 5 major categories (i.e.; biotic, abiotic, morphology, maturity, and composition QTL traits). All overlapped QTL were merged into a single non-redundant QTL, and the redundant QTL were excluded from further analysis. The number of total genes located in the newly merged QTL was manually calculated from the gene/transcript list of *Sorghum bicolor* V3.1 retrieved using the BioMart tool in Phytozome V13 [73]. Moreover, redundant transcripts were filtered out and unique gene IDs were used. Lectins and QTL were mapped in Sorghum chromosomes using MapChart 2.32 [43]. Each QTL was checked for the presence of lectin genes within the identified region based on their physical position.

### Analysis of lectin overrepresentation on QTL

The preferential location of lectins on QTL was statistically analyzed using the Wald test developed by Tsaneva et al. [14]. The significance was determined at *P* values 0.05 and Bonferroni correction 0.025. The calculations were performed by considering the total number of genes (protein-coding transcripts) in the genome (*N*) to be 34,129 (https://phytozome-next.jgi.doe.gov/info/Sbicolor_v3_1_1), and the total number of lectin genes in Sorghum (*s*) is 119 gene.

## Supplementary Information

**Additional file 1:**

**Additional file 2:** 

**Additional file 3:** 

**Additional file 4:** 

**Additional file 5:** 

**Additional file 6:** 

**Additional file 7:** 

## Data Availability

All data generated and analyzed during this study are included in the main article and its supplementary data provided (additional file documents 1–7). Genomic data of sorghum (grian and sweete) are freely accessible and all related UTR links were provided within the article under relevant mentions.
