# Supplementary material for: Genome-wide screening of lectin putative genes from Sorghum bicolor L., distribution in QTLs and a probable implications of lectins in abiotic stress tolerance
Source: BMC Plant Biol. 2022 Aug 13;22:397. doi: 10.1186/s12870-022-03792-6 (PMC9375933; doi:10.1186/s12870-022-03792-6)
Supplement: Supplementary file 1 — Additional file 1: [file 12870_2022_3792_MOESM1_ESM.docx]

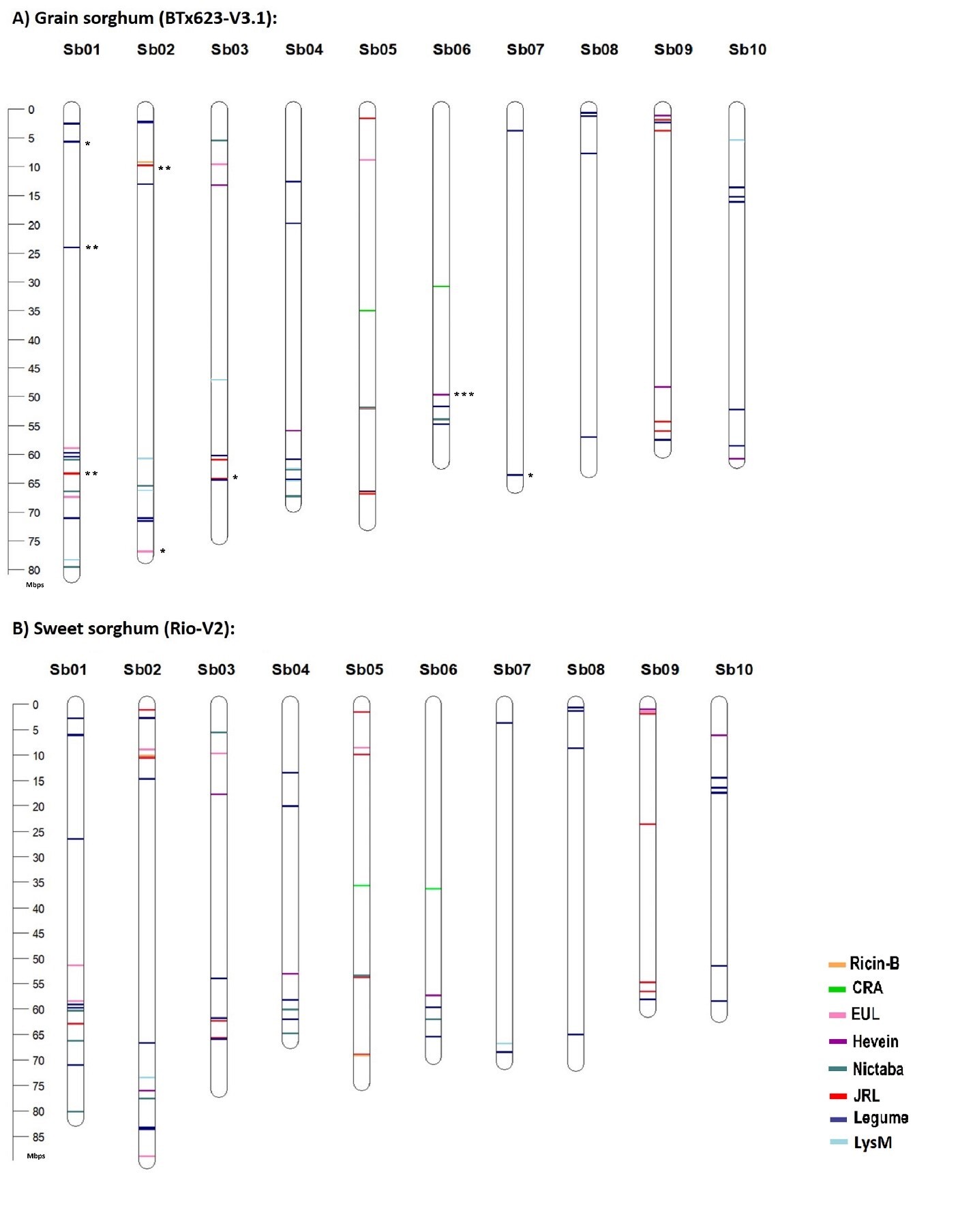


Figure S1: Chromosomal maps of Sorghum bicolor V3.1 and V2.1, illustrating the distribution and abundance of lectin gene homologs in each chromosome. The strikes represent lectin putative genes’ number and location which were tandemly duplicated in grain sorghum genome.


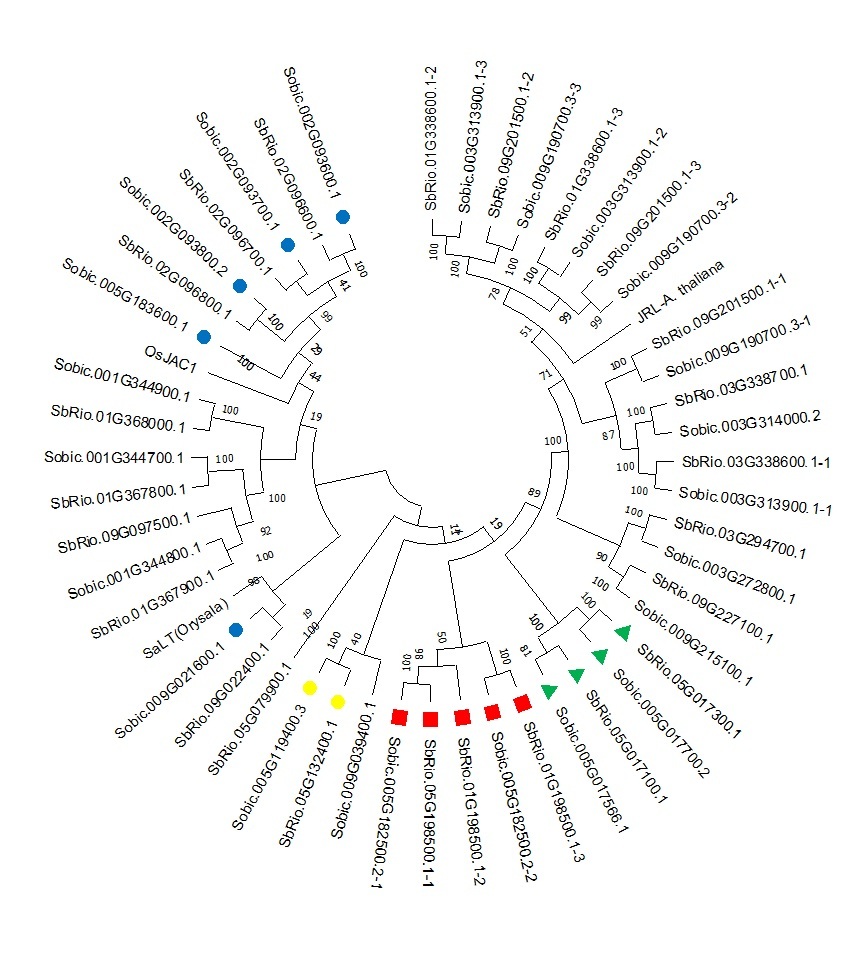


Figure S2: Phylogenetic tree (highest likelihood -5690.01) of Jacalin-related family from both grain and sweet sorghum. Blue circles represent JRL domains that are linked to dirigent domain. Yellow circles are for lectins domain fused with NB-ARC domain. Red squares are JRL domains linked to protein kinase domain. Green triangles are for single JRL domains that possess signal peptide and/or transmembrane domain. Lectins with no designated markers represents mero and hololectin domains. Lectin ID number hyphenated with (1, 2 or 3) are representing the number of lectin domain in multiple domain sequences. Oryza sativa JRL domains from SaLT (Orysala) BAS7201.1 and OsJAC1 ABB51090.1 and JRL-A.thaliana BAL48827.1 from Arabidopsis thaliana were used as a monocot and dicot orthologous groups.
