# Supplementary material for: Genome-wide screening of lectin putative genes from Sorghum bicolor L., distribution in QTLs and a probable implications of lectins in abiotic stress tolerance
Source: BMC Plant Biol. 2022 Aug 13;22:397. doi: 10.1186/s12870-022-03792-6 (PMC9375933; doi:10.1186/s12870-022-03792-6)
Supplement: Supplementary file 3 — Additional file 3: [file 12870_2022_3792_MOESM3_ESM.docx]

Table S1: An overview of QTL related to the major categories and their unique traits (223 traits obtained from the sorghum QTLs atlas). And the distribution of grain sorghum lectin sequences in each category (number and percentage):

|  | | **Abiotic tolerance/ resistance** | | | **Biotic tolerance/ resistance** | | | **Maturity** | | | **Composition** | | | **Morphology** | | |
| --- | --- | --- | --- | --- | --- | --- | --- | --- | --- | --- | --- | --- | --- | --- | --- | --- |
|  |  | **Cold** | **Drought** | **Salinity** | **Fungal** | **Insect** | **Others (Parasitic/bacterial)** | **Days to flowering** | **Photoperiod sensitivity** | **Duration of vegetative stage** | **Leaf** | **Stem** | **Panicle/grain** | **Leaf** | **Panicle/grain** | **Root** |
| **Total QTLs** | | 65 | 76 | 21 | 102 | 26 | 36 | 51 | 2 | 2 | 27 | 61 | 59 | 60 | 25 | 67 |
| **Containing lectin** | **#** | 23 | 25 | 9 | 30 | 19 | 6 | 23 | 2 | 2 | 9 | 14 | 22 | 19 | 13 | 17 |
|  | **%** | **35.4** | **32.9** | **42.9** | **29.4** | **73.1** | **16.7** | **45.1** | **100** | **100** | **33.3** | **22.3** | **37.3** | **31.7** | **52** | **25.4** |
| **All lectins (#119)** | **#** | 98 | 79 | 30 | 64 | 70 | 14 | 84 | 4 | 4 | 23 | 34 | 99 | 70 | 36 | 38 |
|  | **%** | **82** | **66** | **25** | **54** | **59** | **12** | **71** | **3** | **3** | **19** | **29%** | **83** | **59** | **30** | **32** |
| **Ricin-B*** | **#** | 1 | 1 | - | 1 | - | - | 2 | - | - | - | - | 1 | - | - | 1 |
|  | **%** | **50** | **50** | - | **50** | - | - | **100** | - | - | - | - | **50** | - | - | 50 |
| **CRA** | **#** | 2 | 2 | - | - | 1 | - | 2 | - | - | 1 | 1 | 2 | 2 | 1 | 2 |
|  | **%** | **100** | **100** | - | - | **50** | - | **100** | - | - | **50** | **50** | **100** | **100** | **50** | **100** |
| **LysM** | **#** | 8 | 4 | - | 5 | 5 | 2 | 7 | 1 | 1 | 2 | 4 | 7 | 5 | 2 | 2 |
|  | **%** | **100** | **50** | - | 62.5 | **62.5** | 25 | **87.5** | 12.5 | 12.5 | **25** | **50** | **87.5** | **62.5** | **25** | **25** |
| **EUL** | **#** | 5 | 3 | 1 | 2 | 3 | - | 4 | - | - | 1 | - | 4 | 4 | 1 | 2 |
|  | **%** | **62.5** | **37.5** | **12.5** | **25** | **37.5** | - | **50** | - | - | **12.5** | - | **50** | **50** | **12.5** | **25** |
| **Hevein** | **#** | 9 | 5 | 6 | 7 | 7 | - | 9 | - | - | 5 | 6 | 8 | 8 | 5 | 6 |
|  | **%** | **81.8** | **45.5** | **54.5** | **63.6** | **63.6** | - | **81.1** | - | - | **45.5** | **54.5** | **72.7** | **72.7** | **45.5** | **54.5** |
| **Nictaba** | **#** | 8 | 12 | 1 | 5 | 7 | - | 9 | - | - | 2 | 6 | 16 | 7 | 1 | 5 |
|  | **%** | **47.1** | **70.6** | **5.9** | **29.4** | **41.2** | - | **52.9** | - | - | **11.8** | **35.3** | **94.1** | **41.2** | **5.9** | **29.4** |
| **JRL** | **#** | 13 | 13 | 5 | 14 | 10 | - | 12 | - | - | 6 | 3 | 14 | 12 | 2 | 2 |
|  | **%** | **72.2** | **72.2** | **27.8** | **77.8** | **55.6** | - | **66.7** | - | - | **33.3** | **16.7** | **77.8** | **66.7** | **11.1** | **11.1** |
| **Legume** | **#** | 52 | 39 | 17 | 30 | 37 | 12 | 39 | 3 | 3 | 6 | 14 | 47 | 32 | 24 | 18 |
|  | **%** | **98.1** | **73.6** | **32.1** | **56.6** | **69.8** | **22.6** | **73.6** | **5.7** | **5.7** | **11.3** | **26.4** | **88.7** | **60.4** | **45.3** | **34** |

* Percentage per lectin family were calculated based on the total number of lectin homologs within each family.

Table S2-A: QTL related to abiotic traits (susceptibility/tolerance) associated with cold stress. And the distribution of grain sorghum lectin sequences in each sub trait (number and percentage):

|  | | **Abiotic-Cold** | | | | | | | | | | | | | | | | | | | | | |
| --- | --- | --- | --- | --- | --- | --- | --- | --- | --- | --- | --- | --- | --- | --- | --- | --- | --- | --- | --- | --- | --- | --- | --- |
|  |  | **Dry matter growth rate** | **Emergence rate** | **Chlorophyll content** | **Seedling vigor** | **Root length** | **Chlorophyll fluorescence** | **Leaf appearance rate** | **Root biomass** | **Leaf growth rate** | **Shoot fresh weight** | **Germination rate** | **Survival** | **Shoot length** | **Time to emergence** | **Root elongation rate** | **Germination index** | **Early vigor** | **Emergence rate %** | **Transpiration rate** | **stomatal conductance** | **Total dry biomass** |  |
| **Total QTLs** | | 50 | 53 | 75 | 29 | 16 | 78 | 53 | 8 | 44 | 5 | 8 | 16 | 4 | 27 | 2 | 15 | 2 | 7 | 32 | 31 | 5 |  |
| **Containing lectin** | **#** | 13 | 15 | 14 | 3 | 8 | 17 | 16 | 3 | 1 | 3 | 3 | 10 | 2 | 5 | 2 | 1 | 1 | 3 | 2 | 2 | 1 |  |
|  | **%** | **26** | **28.3** | **18.7** | **10.3** | **50** | **21.8** | **30.2** | **37.5** | **2.3** | **60** | **37.5** | **62.5** | **50** | **18.5** | **100** | **6.7** | **50** | **42.9** | **6.3** | **6.5** | **20** |  |
| **All lectins**  (#119) | **#** | 48 | 37 | 30 | 6 | 22 | 46 | 44 | 6 | 1 | 9 | 7 | 30 | 6 | 15 | 6 | 1 | 1 | 5 | 11 | 11 | 2 |  |
|  | **%** | **40** | **31** | **25** | **5** | **18** | **39** | **37** | **5** | **1** | **8** | **6** | **25** | **5** | **13** | **5** | **1** | **1** | **4** | **9** | **9** | **2** |  |
| **Ricin-B*** | **#** | - | 1 | 1 | 1 | - | - | - | - | - | - | - | - | - | - | - | - | - | - | - | - | - |  |
|  | **%** | - | **50** | **50** | **50** | - | - | - | - | - | - | - | - | - | - | - | - | - | - | - | - | - |  |
| **CRA** | **#** | 1 | - | 2 | - | 1 | 1 | - | 1 | - | - | - | - | - | - | - | - | - | - | - | - | - |  |
|  | **%** | **50** | - | **100** | - | **50** | **50** | - | **50** | - | - | - | - | - | - | - | - | - | - | - | - | - |  |
| **LysM** | **#** | 4 | 3 | 1 | 1 | 2 | 7 | 8 | 1 | - | 3 | - | 3 | 1 | 1 | 1 | - | - | 1 | - | - | 1 |  |
|  | **%** | **50** | **37.5** | **12.5** | **12.5** | **25** | **87.5** | **100** | **12.5** |  | **37.5** | - | **37.5** | **12.5** | **12.5** | **12.5** | - | - | **12.5** | - | - | **12.5** |  |
| **EUL** | **#** | 4 | 2 | - | - | 1 | 3 | - | 2 | - | 2 | - | - | - | - | - | - | - | - | - | - | - |  |
|  | **%** | **50** | **25** | - | - | **12.5** | **37.5** | - | **25** | - | **25** | - | - | - | - | - | - | - | - | - | - | - |  |
| **Hevein** | **#** | 3 | 6 | 1 | - | 5 | 2 | 3 | - | - | - | 1 | 5 | 1 | 5 | 5 | - | - | - | 5 | 5 | - |  |
|  | **%** | **27.3** | **54.5** | **9.1** | - | **45.5** | **18.2** | **27.3** | - | - | - | **9.1** | **45.5** | **9.1** | **45.5** | **45.5** | - | - | - | **45.5** | **45.5** | - |  |
| **Nictaba** | **#** | 5 | 3 | - | - | 2 | 6 | 7 | 1 | - | 1 | - | - | - | - | - | 1 | 1 | - | - | - | - |  |
|  | **%** | **29.4** | **17.6** | - | - | **11.7** | **35.3** | **41.2** | **5.9** | - | **5.9** | - | - | - | - | - | **5.9** | **5.9** | - | - | - | - |  |
| **JRL** | **#** | 11 | 6 | 2 | - | 1 | 3 | 5 | - | - | - | 2 | - | 3 | - | - | - | - | 2 | - | - | - |  |
|  | **%** | **61.1** | **33.3** | **11.1** | - | **5.6** | **16.7** | **27.8** | - | - | - | **11.1** | - | **16.7** | - | - | - | - | **11.1** | - | - | - |  |
| **Legume** | **#** | 17 | 13 | 23 | 4 | 10 | 25 | 21 | 1 | 1 | 3 | 4 | 20 | 1 | 9 | - | - | - | 2 | 6 | 6 | 1 |  |
|  | **%** | **32.1** | **24.5** | **43.4** | **7.5** | **18.9** | **47.2** | **39.6** | **1.9** | **1.9** | **5.7** | **7.5** | **37.7** | **1.9** | **16.9** | - | - | - | **3.8** | **11.3** | **11.3** | **1.9** |  |

* Percentage per lectin family were calculated based on the total number of lectin homologs within each family.

Table S2-B: QTL related to abiotic traits (susceptibility/tolerance) associated with drought and salinity stresses. And the distribution of grain sorghum lectin sequences in each sub trait (number and percentage):

|  | | **Abiotic-Drought** | | | | | | | | | | | | | | | **Abiotic-Salinity** | | | | | | | |  |
| --- | --- | --- | --- | --- | --- | --- | --- | --- | --- | --- | --- | --- | --- | --- | --- | --- | --- | --- | --- | --- | --- | --- | --- | --- | --- |
|  |  | **Stay-green** | **Green leaf area** | **Age-related leaf senescence** | **Chlorophyll content** | **CO2 assimilation ratio** | **Transpiration** | **Leaf area** | **Height (plant height)** | **Dry leaf weight** | **Grain number** | **Grain yield** | **Grain weight** | **Stomatal density** | **Total number of green leaves** | **Transpiration ratio (A: E)** | **Germination rate (%)** | **Shoot dry weight** | **Shoot height** | **Root fresh weight** | **Fresh biomass** | **Total fresh weight** | **Germination vigor** | **Total dry weight** | |
| **Total QTLs** | | 13 | 22 | 3 | 29 | 3 | 2 | 3 | 4 | 2 | 1 | 6 | 2 | 2 | 6 | 1 | 5 | 5 | 4 | 3 | 2 | 3 | 3 | 2 | |
| **Containing lectin** | **#** | 5 | 9 | 1 | 9 | 1 | 1 | 3 | 1 | 1 | 1 | 3 | 1 | 1 | 3 | 1 | 2 | 2 | 1 | 1 | 1 | 2 | 2 | 1 | |
|  | **%** | **38.5** | **40.9** | **33.3** | **31** | **33.3** | **50** | **100** | **25** | **50** | **100** | **50** | **50** | **50** | **50** | **100** | **40** | **40** | **25** | **33.3** | **50** | **66.7** | **66.7** | **50** | |
| **All lectins**  (#119) | **#** | 12 | 34 | 1 | 17 | 1 | 1 | 10 | 1 | 3 | 2 | 4 | 1 | 2 | 11 | 2 | 7 | 5 | 8 | 5 | 5 | 5 | 4 | 1 | |
|  | **%** | **10** | **29** | **1** | **14** | **1** | **1** | **8** | **1** | **3** | **2** | **3** | **1** | **2** | **9** | **2** | **6** | **4** | **7** | **4** | **4** | **4** | **3** | **1** | |
| **Ricin-B*** | **#** | - | - | - | 1 | - | - | - | - | - | - | - | - | - | - | - | - | - | - | - | - | - | - | - | |
|  | **%** | - | - | - | **50** | - | - | - | - | - | - | - | - | - | - | - | - | - | - | - | - | - | - | - | |
| **CRA** | **#** | 1 | 1 | - | - | - | - | - | - | - | - | - | - | - | - | - | - | - | - | - | - | - | - | - | |
|  | **%** | **50** | **50** | - | - | - | - | - | - | - | - | - | - | - | - | - | - | - | - | - | - | - | - | - | |
| **LysM** | **#** | 1 | 2 | - | 2 | - | - | - | - | - | - | - | - | - | - | - | - | - | - | - | - | - | - | - | |
|  | **%** | **12.5** | **25** | - | **25** | - | - | - | - | - | - | - | - | - | - | - | - | - | - | - | - | - | - | - | |
| **EUL** | **#** | 1 | - | 1 | - | 1 | 1 | 2 | - | - | - | - | - | - | - | - | 1 | - | - | - | - | - | - | - | |
|  | **%** | **12.5** | - | **12.5** | - | **12.5** | **12.5** | **25** | - | - | - | - | - | - | - | - | **12.5** | - | - | - | - | - | - | - | |
| **Hevein** | **#** | 5 | 6 | - | - | - | - | - | - | - | - | - | - | - | - | - | - | 1 | 1 | 5 | 5 | - | - | - | |
|  | **%** | **45.5** | **54.5** | - | - | - | - | - | - | - | - | - | - | - | - | - | - | **9.1** | **9.1** | **45.5** | **45.5** | - | - | - | |
| **Nictaba** | **#** | 2 | 8 | - | 1 | - | - | - | 1 | - | - | 1 | - | - | - | - | 1 | - | - | - | - | - | - | - | |
|  | **%** | **11.8** | **47.1** | - | **5.9** | - | - | - | **5.9** | - | - | **5.9** | - | - | - | - | **5.9** | - | - | - | - | - | - | - | |
| **JRL** | **#** | 1 | 5 | - | 5 | - | - | - | - | 3 | 2 | - | - | - | - | - | - | 2 | - | - | - | 2 | 3 | - | |
|  | **%** | **5.6** | **27.8** | **-** | **27.8** | - | - | - | - | **16.7** | **27.8** | - | - | - | - | - | - | **27.8** | - | - | - | **27.8** | **16.7** | - | |
| **Legume** | **#** | 1 | 12 | - | 8 | - | - | 8 | - | - | - | 3 | 1 | 2 | 11 | 2 | 5 | 2 | 7 | - | - | 3 | 1 | 1 | |
|  | **%** | **1.9** | **22.6** | - | **15.1** | - | - | **15.1** | - | - | - | **5.6** | **1.9** | **3.8** | **20.8** | **3.8** | **9.4** | **3.8** | **13.2** | - | - | **5.6** | **1.9** | **1.9** | |

* Percentage per lectin family were calculated based on the total number of lectin homologs within each family.

Table S3: QTL related to biotic traits (susceptibility/resistance) associated with fungal, insect and parasitic infection. And the distribution of grain sorghum lectin sequences in each sub trait (number and percentage):

|  | | **Biotic-Fungal** | | | | | | | | | | | | |  | | **Biotic-Insect** | | | | | | | **Parasitic** |
| --- | --- | --- | --- | --- | --- | --- | --- | --- | --- | --- | --- | --- | --- | --- | --- | --- | --- | --- | --- | --- | --- | --- | --- | --- |
|  |  | **Resistance to anthracnose (Colletotrichum sublineolum)** | **Resistance to downy mildew: susceptibility** | **Midge resistance (antixenosis)** | **Ergot resistance (pollen viability)** | **Rust resistance** | **Resistance to head smut** | **Charcoal rot disease severity** | **Stalk rot (% lodging)** | **Grain mould** | **Major lesion length (Macrophomina phaseolina)** | **Grain mould resistance** | **Ergot resistance (% ergot infection)** | **Ergot resistance (pollen quantity)** | **Downy mildew resistance** | **Relative major lesion length (Fusarium thapsinum)** | **Green bug resistance** | **Rice weevil resistance** | **Head bug resistance (1-9)** | **Shoot fly resistance (trichome density on lower leaf surface)** | **Shoot fly resistance (seedling vigour)** | **Shoot fly resistance (glossiness)** | **Shoot fly resistance (mean deadheart % on 28 DAE)** | **Resistance to the parasitic weed, Striga** |
| **Total QTLs** | | 23 | 1 | 2 | 3 | 66 | 16 | 3 | 2 | 10 | 4 | 3 | 9 | 5 | 7 | 2 | 12 | 6 | 7 | 16 | 4 | 4 | 4 | 28 |
| **Containing lectin** | **#** | 7 | 1 | 1 | 2 | 18 | 2 | 1 | 2 | 3 | 2 | 1 | 5 | 2 | 1 | 1 | 9 | 4 | 5 | 10 | 1 | 1 | 1 | 6 |
|  | **%** | **30.4** | **100** | **50** | **66.7** | **27.3** | **12.5** | **33.3** | **100** | **30** | **50** | **33.3** | **55.6** | **40** | **14.3** | **50** | **75** | **66.7** | **71.4** | **62.5** | **25** | **25** | **25** | **21.4** |
| **All lectins**  (#119) | **#** | 10 | 1 | 1 | 3 | 25 | 6 | 2 | 3 | 10 | 3 | 3 | 13 | 12 | 6 | 1 | 41 | 11 | 12 | 24 | 5 | 5 | 7 | 14 |
|  | **%** | **8** | **1** | **1** | **3** | **21** | **5** | **2** | **3** | **8** | **3** | **3** | **11** | **10** | **5** | **1** | **34** | **9** | **10** | **20** | **4** | **4** | **6** | **12** |
| **Ricin-B*** | **#** | 1 | - | - | - | - | - | - | - | - | - | - | - | - | - | - | - | - | - | - | - | - | - | - |
|  | **%** | **50** | - | - | - | - | - | - | - | - | - | - | - | - | - | - | - | - | - | - | - | - | - | - |
| **CRA** | **#** | - | - | - | - | - | - | - | - | - | - | - | - | - | - | - | 1 | 1 | - | - | - | - | - | - |
|  | **%** | - | - | - | - | - | - | - | - | - | - | - | - | - | - | - | **50** | **50** | - | - | - | - | - | - |
| **LysM** | **#** | - | - | - | - | 4 | - | - | - | - | - | - | - | - | - | - | 2 | 1 | 1 | - | - | - | - | 1 |
|  | **%** | - | - | - | - | **50** | - | - | - | - | - | - | - | - | - | - | **25** | **12.5** | **12.5** | - | - | - | - | **12.5** |
| **EUL** | **#** | - | 1 | 1 | 1 | 1 | - | - | - | - | - | - | - | - | - | - | 3 | - | 1 | 2 | - | - | - | - |
|  | **%** | - | **12.5** | **12.5** | **12.5** | **12.5** | - | - | - | - | - | - | - | - | - | - | **37.5** | - | **12.5** | **25** | - | - | - | - |
| **Hevein** | **#** | 1 | - | - | - | 2 | 5 | - | - | - | - | - | - | - | - | - | 6 | - | - | 5 | 5 | 5 | - | - |
|  | **%** | **9.1** | - | - | - | 18.2 | **45.5** | - | - | - | - | - | - | - | - | - | 54.5 | - | - | **45.5** | **45.5** | **45.5** | - | - |
| **Nictaba** | **#** | 2 | - | - | - | 4 | - | 1 | - | - | - | - | - | - | - | - | 6 | - | 1 | 1 | - | - | - | - |
|  | **%** | **11.7** | - | - | - | **23.5** | - | **5.9** | - | - | - | - | - | - | - | - | **35.3** | - | **5.9** | **5.9** | - | - | - | - |
| **JRL** | **#** | 3 | - | - | - | 7 | - | 1 | - | - | 1 | 3 | - | - | - | - | 7 | 3 | 1 | - | - | - | - | 1 |
|  | **%** | **16.7** | - | - | - | **38.9** | - | **5.5** | - | - | **5.5** | **16.7** | - | - | - | - | **38.9** | **16.7** | **5.5** | - | - | - | - | **5.5** |
| **Legume** | **#** | 3 | - | - | 2 | 7 | 1 | - | 3 | 10 | 2 | - | 14 | 12 | 6 | 1 | 15 | 6 | 8 | 16 | - | - | 7 | 12 |
|  | **%** | **5.7** | - | - | **3.8** | **13.2** | **1.9** | - | **5.7** | **18.9** | **3.8** | - | **26.4** | **22.6** | **11.3** | **1.9** | **28.3** | **11.3** | **15.1** | **30.2** | - | - | **13.2** | **22.6** |

* Percentage per lectin family were calculated based on the total number of lectin homologs within each family.

Table S4-A: QTL related to maturity and composition traits associated with leaf and stem. And the distribution of grain sorghum lectin sequences in each sub trait (number and percentage):

|  | | **Maturity** | | | **Composition- Leaf** | | | **Composition-Stem** | | | | | | |
| --- | --- | --- | --- | --- | --- | --- | --- | --- | --- | --- | --- | --- | --- | --- |
|  |  | **Days to flowering** | **Photoperiod sensitivity** | **Duration of Vegetative stage** | **Leaf chlorophyll content** | **Leaf hydrolysis yield potential** | **Leaf cellulose** | **Stem hydrolysis yield potential** | **Neutral detergent fibre** | **Acid detergent fibre** | **Cellulose content** | **Hemicellulose content** | **Phosphorus content** | **Lignin content** |
| **Total QTLs** | | 51 | 2 | 2 | 17 | 5 | 1 | 6 | 25 | 11 | 15 | 8 | 3 | 3 |
| **Containing lectin** | **#** | 23 | 2 | 2 | 6 | 2 | 1 | 4 | 2 | 5 | 7 | 2 | 1 | 1 |
|  | **%** | **45.1** | **100** | **100** | **35.3** | **40** | **100** | **66.7** | **8** | **45.5** | **46.7** | **25** | **33.3** | **33.3** |
| **All lectins**  (#119) | **#** | 84 | 4 | 4 | 14 | 4 | 3 | 8 | 6 | 6 | 20 | 9 | 1 | 1 |
|  | **%** | **71** | **3** | **3** | **12** | **3** | **3** | **7** | **5** | **5** | **17** | **8** | **1** | **1** |
| **Ricin-B*** | **#** | 2 | - | - | - | - | - | - | - | - | - | - | - | - |
|  | **%** | **100** | - | - | - | - | - | - | - | - | - | - | - | - |
| **CRA** | **#** | 2 | - | - | 1 | - | - | 1 | - | - | - | - | - | - |
|  | **%** | **100** | - | - | **50** | - | - | **50** | - | - | - | - | - | - |
| **LysM** | **#** | 7 | 1 | 1 | **2** | - | - | **-** | - | 3 | 2 | - | - | - |
|  | **%** | **87.5** | **12.5** | **12.5** | **25** | - | - | **-** | - | **37.5** | **25** | - | - | - |
| **EUL** | **#** | 4 | - | - | 1 | - | - | - | - | - | - | - | - | - |
|  | **%** | **50** | - | - | **12.5** | - | - | - | - | - | - | - | - | - |
| **Hevein** | **#** | 9 | - | - | - | - | - | 1 | 5 | - | - | - | - | - |
|  | **%** | **81.1** | - | - | - | - | - | **9.1** | **45.5** | - | - | - | - | - |
| **Nictaba** | **#** | 9 | - | - | 2 | - | - | 3 | 1 | 1 | 1 | - | - | - |
|  | **%** | **52.9** | - | - | **11.7** | - | - | **17.6** | **5.9** | **5.9** | **5.9** | - | - | - |
| **JRL** | **#** | 12 | - | - | 2 | 3 | 2 | 1 | - | 1 | 2 | - | - | - |
|  | **%** | **66.7** | - | - | **11.1** | **16.7** | **11.1** | **5.6** | - | **5.6** | **11.1** | - | - | - |
| **Legume** | **#** | 39 | 3 | 3 | 4 | 1 | 1 | 2 | - | 1 | 13 | 9 | 1 | 1 |
|  | **%** | **73.6** | **5.7** | **5.7** | **7.5** | **1.9** | **1.9** | 3.7 | - | **1.9** | **24.5** | **16.9** | **1.9** | **1.9** |

* Percentage per lectin family were calculated based on the total number of lectin homologs within each family.

Table S4-B: QTL related to composition traits associated with panicle/grain, and root. And the distribution of grain sorghum lectin sequences in each sub trait (number and percentage):

|  | | **Composition- Panicle/grain** | | | | | | | | | | | | | | | | | | | | | |
| --- | --- | --- | --- | --- | --- | --- | --- | --- | --- | --- | --- | --- | --- | --- | --- | --- | --- | --- | --- | --- | --- | --- | --- |
|  |  | **Grain color** | **Amino Acid content** | **Kernel flouriness** | **Grain morphology** | **Amylose content** | **Grain element concentration** | **Tannin content** | **Carotenoid content of endosperm** | **3-deoxyanthocyanidins** | **Proanthocyanidins** | **Grain fat content** | **Protein content** | **Starch** | **Total Flavenoid content** | **Grain fibre content** | **Kernel hardness** | **Corneous endosperm** | **Embryo size** | **Polyphenol content** | **To Characteristics** | **Protein digestibility** |  |
| **Total QTLs** | | 40 | 163 | 13 | 3 | 34 | 112 | 24 | 33 | 28 | 49 | 13 | 16 | 21 | 16 | 7 | 8 | 2 | 4 | 14 | 3 | 2 |  |
| **Containing lectin** | **#** | 12 | 14 | 11 | 2 | 9 | 14 | 3 | 8 | 3 | 11 | 8 | 6 | 6 | 2 | 6 | 3 | 2 | 3 | 2 | 1 | 1 |  |
|  | **%** | **30** | **8.5** | **84.6** | **66.7** | **26.4** | **12.5** | **12.5** | **24.2** | **10.7** | **22.4** | **61.5** | **37.5** | **28.6** | **12.5** | **85.7** | **37.5** | **100** | **25** | **14.3** | **33.3** | **50** |  |
| **All lectins**  (#119) | **#** | 38 | 34 | 29 | 2 | 34 | 14 | 3 | 8 | 4 | 13 | 21 | 25 | 14 | 15 | 17 | 10 | 3 | 4 | 2 | 3 | 2 |  |
|  | **%** | **32** | **29** | **24** | **2** | **29** | **12** | **3** | **7** | **3** | **11** | **18** | **21** | **12** | **13** | **14** | **8** | **3** | **3** | **2** | **3** | **2** |  |
| **Ricin-B*** | **#** | 1 | 1 | 1 | 1 | 1 | 1 | - | - | - | - | - | - | - | - | 1 | - | - | - | - | - | - |  |
|  | **%** | **50** | **50** | **50** | **50** | **50** | **50** | - | - | - | - | - | - | - | - | **50** | - | - | - | - | - | - |  |
| **CRA** | **#** | 2 | - | - | - | 1 | 1 | 1 | 1 | 1 | 2 | 1 | 1 | - | - | - | - | - | - | - | - | - |  |
|  | **%** | **100** | - | - | - | **50** | **50** | **50** | **50** | **50** | **100** | **50** | **50** | - | - | - | - | - | - | - | - | - |  |
| **LysM** | **#** | **6** | 2 | 2 | 1 | **4** | **3** | **-** | **-** | **1** | **2** | **-** | **2** | 2 | - | 3 | - | - | - | 1 | 1 | - |  |
|  | **%** | **75** | **25** | **25** | **12.5** | **50** | **37.5** | **-** | **-** | **12.5** | **25** | **-** | **25** | **25** | - | **37.5** | - | - | - | **12.5** | **12.5** | - |  |
| **EUL** | **#** | 4 | 3 | 2 | - | 1 | - | - | - | - | - | 2 | - | - | - | - | - | - | - | - | - | - |  |
|  | **%** | **50** | **37.5** | **25** | - | **12.5** | - | - | - | - | - | **25** | - | - | - | - | - | - | - | - | - | - |  |
| **Hevein** | **#** | - | 1 | 1 | - | 2 | - | - | - | 2 | 4 | 5 | 1 | 1 | - | 1 | - | - | - | - | - | - |  |
|  | **%** | - | **9.1** | **9.1** | - | **18.2** | - | - | - | **18.2** | **36.4** | **45.5** | **9.1** | **9.1** | - | **9.1** | - | - | - | - | - | - |  |
| **Nictaba** | **#** | 14 | 9 | 3 | - | 9 | 1 | 2 | 2 | - | 3 | 1 | - | - | 8 | 1 | - | - | - | - | 1 | - |  |
|  | **%** | **82.4** | **52.9** | **17.6** | - | **52.9** | **5.8** | **11.7** | **11.7** | - | **17.6** | **5.8** | - | - | **47.1** | **5.8** | - | - | - | - | **5.8** | - |  |
| **JRL** | **#** | 4 | 3 | 3 | - | 4 | 4 | - | - | - | - | 6 | 2 | 4 | - | 2 | 2 | - | - | - | - | - |  |
|  | **%** | **22.2** | **16.7** | **16.7** | - | **22.2** | **22.2** | - | - | - | - | **33.3** | **11.1** | **22.2** | - | **11.1** | **11.1** | - | - | - | - | - |  |
| **Legume** | **#** | 7 | 15 | 18 | - | 11 | 4 | - | 5 | - | 2 | 5 | 19 | 7 | 7 | 9 | 8 | 3 | 4 | 1 | 1 | 2 |  |
|  | **%** | **13.2** | **28.3** | **33.9** | - | **20.7** | **7.5** | - | **9.4** | - | **3.8** | **9.4** | **35.8** | **13.2** | **13.2** | **16.9** | **15.1** | **5.7** | **7.5** | **1.9** | **1.9** | **3.8** |  |

* Percentage per lectin family were calculated based on the total number of lectin homologs within each family.

Table S5: QTL related to morphological traits associated with leaf, panicle/grain, and root. And the distribution of grain sorghum lectin sequences in each sub trait (number and percentage):

|  | | **Morphology-Leaf** | | | | | | | | | | | | | **Morphology-Panicle** | | **Morphology-Root** | | | | | | | |
| --- | --- | --- | --- | --- | --- | --- | --- | --- | --- | --- | --- | --- | --- | --- | --- | --- | --- | --- | --- | --- | --- | --- | --- | --- |
|  |  | Phyllochron | Leaf width | Leaf angle | Leaf Length Increasing Rate | Leaf length | Epicuticular wax | Leaf width increasing Rate | flag leaf length | flag leaf width | Flag leaf angle | Leaf curve | Leaf pitch | Leaf sheath color | Panicle length | Peduncle length | Root biomass | Network solidity | Root angle | Brace root | Root to shoot ratio | Root dry weight | Root volume |  |
| **Total QTLs** | | 6 | 15 | 23 | 4 | 11 | 15 | 6 | 4 | 11 | 2 | 5 | 2 | 2 | 24 | 7 | 7 | 4 | 5 | 2 | 4 | 11 | 2 |  |
| **Containing lectin** | **#** | 4 | 11 | 6 | 2 | 7 | 4 | 2 | 2 | 3 | 2 | 3 | 1 | 1 | 12 | 3 | 1 | 1 | 4 | 1 | 3 | 5 | 2 |  |
|  | **%** | **66.7** | **73.3** | **26.1** | **50** | **63.6** | **26.7** | **33.3** | **50** | **27.3** | **100** | **60** | **50** | **50** | **50** | **42.9** | **14.3** | **25** | **80** | **50** | **75** | **45.5** | **100** |  |
| **All lectins**  (#119) | **#** | 10 | 36 | 10 | 3 | 19 | 5 | 2 | 9 | 8 | 10 | 4 | 2 | 2 | 32 | 6 | 1 | 1 | 7 | 1 | 3 | 7 | 2 |  |
|  | **%** | **8** | **30** | **8** | **3** | **16** | **4** | **2** | **8** | **7** | **8** | **3** | **2** | **2** | **27** | **5** | **1** | **1** | **6** | **1** | **3** | **6** | **2** |  |
| **Ricin-B*** | **#** | - | - | - | - | - | - | - | - | - | - | - | - | - | - | - | 1 | - | - | - | - | - | - |  |
|  | **%** | - | - | - | - | - | - | - | - | - | - | - | - | - | - | - | **50** | - | - | - | - | - | - |  |
| **CRA** | **#** | 1 | 1 | 1 | - | - | - | - | - | - | - | - | - | - | 1 | - | - | - | 1 | 1 | - | - | - |  |
|  | **%** | **50** | **50** | **50** | - | - | - | - | - | - | - | - | - | - | **50** | - | - | - | **50** | **50** | - | - | - |  |
| **LysM** | **#** | - | 2 | 1 | 1 | 4 | 1 | 1 | 1 | 1 | 1 | 1 | 1 | 1 | 2 | - | - | - | - | - | - | - | - |  |
|  | **%** | **-** | **25** | **12.5** | **12.5** | **50** | **12.5** | **12.5** | **12.5** | **12.5** | **12.5** | **12.5** | **12.5** | **12.5** | **25** | - | - | - | **-** | **-** | - | - | - |  |
| **EUL** | **#** | 2 | 1 | 2 | 1 | 1 | - | - | - | - | - | - | - | - | 1 | - | - | - | - | - | - | - | - |  |
|  | **%** | **25** | **12.5** | **25** | **12.5** | **12.5** | - | - | - | - | - | - | - | - | **12.5** | - | - | - | - | - | - | - | - |  |
| **Hevein** | **#** | - | 5 | 1 | - | - | 2 | - | - | - | - | - | - | - | 5 | - | - | - | - | - | 1 | - | - |  |
|  | **%** | - | **45.5** | **9.1** | - | - | **18.2** | - | - | - | - | - | - | - | **45.5** | - | - | - | - | - | **9.1** | - | - |  |
| **Nictaba** | **#** | 3 | 3 | 1 | 1 | 2 | - | - | - | - | 1 | - | - | - | - | 2 | - | - | 3 | 1 | 3 | 3 | - |  |
|  | **%** | **17.6** | **17.6** | **5.8** | **5.8** | **11.8** | - | - | - | - | **5.8** | - | - | - | - | **11.8** | - | - | **17.6** | **5.8** | **17.6** | **17.6** | - |  |
| **JRL** | **#** | 1 | 11 | - | - | 3 | 1 | - | - | - | - | - | - | - | - | 2 | - | - | 1 | - | - | 2 | - |  |
|  | **%** | **5.5** | **61.1** | - | - | **16.6** | **5.5** | - | - | - | - | - | - | - | - | **11.1** | - | - | **5.5** | - | - | **11.1** | - |  |
| **Legume** | **#** | 3 | 12 | 3 | - | 9 | 1 | 1 | 8 | 7 | 8 | 3 | 1 | 1 | 23 | 2 | - | 1 | 2 | - | 1 | 2 | 2 |  |
|  | **%** | **5.6** | **22.6** | **5.6** | - | **16.9** | **1.9** | **1.9** | **15.1** | **13.2** | **15.1** | **5.6** | **1.9** | **1.9** | **43.4** | **3.7** | - | **1.9** | **3.7** | **-** | **1.9** | **3.7** | **3.7** |  |

* Percentage per lectin family were calculated based on the total number of lectin homologs within each family.
